# ROV observations reveal infection dynamics of gill parasites in midwater cephalopods

**DOI:** 10.1038/s41598-022-11844-y

**Published:** 2022-05-18

**Authors:** Vanessa I. Stenvers, Rob E. Sherlock, Kim R. Reisenbichler, Bruce H. Robison

**Affiliations:** 1 GEOMAR, Helmholtz Centre for Ocean Research Kiel, Düsternbrooker Weg 20, 24105 Kiel, Germany; 2 Department of Invertebrate Zoology, National Museum of Natural History, Smithsonian Institution, Washington, DC 20013 USA; 3 Monterey Bay Aquarium Research Institute, 7700 Sandholdt Road, Moss Landing, CA 95039-9644 USA

**Keywords:** Biodiversity, Ecosystem ecology, Ecology, Ecology, Coevolution, Phylogenetics

## Abstract

Gill parasites of coleoid cephalopods are frequently observed during remotely operated vehicle (ROV) dives in the Monterey Submarine Canyon. However, little knowledge exists on the identity of the parasite species or their effects on the cephalopod community. With the help of ROV-collected specimens and in situ footage from the past 27 years, we report on their identity, prevalence and potential infection strategy. Gill parasites were genetically and morphologically identified from collected specimens of *Chiroteuthis calyx*, *Vampyroteuthis infernalis* and *Gonatus* spp*. *In situ prevalence was estimated from video footage for *C. calyx*, *Galiteuthis* spp., *Taonius* spp. and *Japetella diaphana*, enabled by their transparent mantle tissue. The most common parasite was identified as *Hochbergia* cf. *moroteuthensis*, a protist of unresolved taxonomic ranking. We provide the first molecular data for this parasite and show a sister group relationship to the dinoflagellate genus *Oodinium*. *Hochbergia* cf. *moroteuthensis* was most commonly observed in adult individuals of all species and was sighted year round over the analyzed time period. In situ prevalence was highest in *C. calyx* (75%), followed by *Galiteuthis* spp. (29%), *Taonius* spp. (27%) and *J. diaphana* (7%). A second parasite, not seen on the in situ footage, but occurring within the gills of *Gonatus berryi* and *Vampyroteuthis infernalis*, could not be found in the literature or be identified through DNA barcoding. The need for further investigation is highlighted, making this study a starting point for unravelling ecological implications of the cephalopod-gill-parasite system in deep pelagic waters.

## Introduction

Despite parasitism being one of the most common and successful life-strategies on Earth, parasites maintain a rather infamous reputation^1–3^. Defined as living at the expense of their hosts, it is not surprising that little appreciation is fostered for parasitic creatures. Even if a parasitic infection does not result in disease, the parasite will divert host-resources for its own use and thereby lower host fitness^3^. Depending on parasite prevalence and virulence, physiological effects can range from reducing host growth to infertility, or mortality in severe cases^3,4^. Subsequently, at the level of the individual being parasitized, the infection is certainly less than ideal. When approaching the matter at the population level, however, the parasite stigma appears largely unjustified. Increasing evidence points to a key role for parasites in ecosystem functioning and identifies them as important agents in maintaining biodiversity^4,5^. Not only can a host-parasite relationship result in selection for a stronger gene pool (i.e. to better resist or infect one another through co-evolution), but also facilitate the co-existence of species by limiting strongly competitive organisms. Moreover, in some cases, parasites have been shown to provide direct benefits for their hosts such as the diversion and accumulation of environmental toxins, or prevention of more harmful parasite-infections by means of competitive exclusion^4–6^.

Knowledge of parasite ecology is predominantly derived from terrestrial or coastal studies, yet the largest habitat on Earth, the ocean’s midwater zone, is generally excluded^7,8^. The deep pelagic ocean, stretching below 200 m, lies far beyond the reach of conventional study methods like SCUBA, and has only recently been influenced by advances in technology, survey and sampling equipment^9,10^. The little, but steadily growing research available, suggests that the midwater is no exception when it comes to parasitic infections. Since there are few places to hide and few counterparts of benthic substrate, parasitic lifestyles offer an alternative. The bodies of many gelatinous animals (e.g. pelagic tunicates, gossamer worms) have been found to facilitate ontogenetic development in a variety of parasites, by acting as breeding grounds, nurseries or food resources^11–13^. For example, the exclusively pelagic hyperiid amphipods often parasitize gelatinous zooplankton during some stage of their life cycles^14^. Nevertheless, since most parasites are inconspicuous and may have complex life-histories^8^, much remains to be learned about parasitic relationships in the midwater zone.

A host-parasite system that offers interesting research opportunities is the coleoid cephalopods (i.e. squid, cuttlefish and octopods) and their parasites. Coleoids are common components of the mesopelagic community^15,16^, with the ca. 326 million year-old fossil record^17^ suggesting ample time for parasites to have optimized their infection strategies. Of the few cephalopods studied (i.e. less than twenty-five percent of all species described), almost all mature individuals were shown to possess some sort of parasitic symbiont^18,19^. In addition, the taxonomic diversity of cephalopod parasites is similar to the diversity of parasites found in fishes, varying from viruses, bacteria, protozoans, mesozoans, platyhelminths, acanthocephalans, nematodes, and annelids to crustaceans^7,18–20^. In spite of this, as with other parasite studies, most research efforts are focused on shallow coastal species^7^, leaving us, at best, with an educated guess about cephalopod-parasite relationships in the deeper ocean.

Understanding host-parasite systems is important in the light of the increasing amount of human-induced environmental changes, which include rapid warming of the ocean’s climate and exposure of its inhabitants to a variety of abiotic and biotic pressures (e.g. ocean acidification, deoxygenation and changes in food web structure^21^). In the case where host and parasite have co-evolved, shifting environmental conditions could easily offset their relationship^22,23^. As a result, hosts could become more sensitive to infections or be exposed to invasive parasites for which they do not possess sufficient defense mechanisms^23^. Furthermore, parasite or host populations could change in density, altering selective pressures on the community^22^. The ecological implications of such changes could be large, since coleoid cephalopods form key links in marine food webs that cross multiple trophic levels and impact commercial fisheries^24,25^. Nevertheless, to understand in what direction a cephalopod-parasite system might shift with changing environmental conditions, the relationship needs to be characterized first.

In this study we aim to provide a better understanding of the parasites present in several pelagic cephalopods, as documented by remotely operated vehicles (ROVs) in the Monterey Submarine Canyon (Monterey Bay, CA, USA). The transparent tissue in some of these coleoids allows for unique insights into parts of their interior^26^. When illuminated by the bright lights of an ROV, internal structures, including gills and macroscopic parasites, become visible. The main goals here are to (*i*) identify these parasites with help of DNA barcoding, (*ii*) determine their prevalence by using in situ footage from the Monterey Bay Aquarium Research Institute (MBARI) video database and (*iii*) infer a potential infection strategy based on the latter results. This study serves as a starting point to gain a broader ecological understanding of the cephalopod-gill-parasite system in a deep pelagic community.

## Materials and methods

### Sample collection and parasite removal

To identify the parasites observed during ROV dives morphologically and genetically, three coleoid cephalopods within the orders Oegopsida and Vampyromorpha were sampled during June and July of 2019 in the Monterey Submarine Canyon: *Chiroteuthis calyx* Young, 1972 (n = 3, including one paralarva), *Gonatus* spp*.* (comprising *Gonatus berryi* Naef, 1923, *Gonatus onyx* Young, 1972 and *Gonatus* sp*.*; n = 8, including two paralarvae) and *Vampyroteuthis infernalis* Chun, 1903 (n = 1). Specimens were collected using 7.5 L static detritus, and variable-flow suction samplers^9^, mounted on MBARI’s ROVs *Doc Ricketts* and *Ventana,* operated from the research vessels *Western Flyer* and *Rachel Carson*, respectively. All specimens in this study were collected under valid US State and Federal Scientific Collecting Permits. Although ethics approval is not needed for cephalopods in the United States of America, special effort was made to minimize suffering and distress in caught specimens^27^. The *Chiroteuthis calyx* and *Gonatus* spp*.* specimens, if alive after the ROV ascent, were humanely killed by an incision between the eyes to destroy the brain. Due to its large size and fragile tissue, the *V. infernalis* specimen was anaesthetized by adding an overdose of MgCl_2_ to the sample container before destroying the brain. For all specimens, a ventral incision was made in the mantle to dissect the gills, while using a light microscope to search for parasites. Parasites with similar morphology from one gill were pooled in Eppendorf tubes and frozen in liquid nitrogen to be stored at – 80 °C until the DNA extraction. Parasites from the other gill were preserved in 3.5% Glutaraldehyde for later reference. The remainder of the animal was preserved in 5% formaldehyde and seawater solution.

### Prevalence and infection intensity

To get estimates of parasite prevalence and infection intensity, archived in situ footage of the Oegopsid squid *Chiroteuthis calyx*, *Galiteuthis* spp*.* Joubin, 1898, *Taonius* spp. Steenstrup, 1861 and the octopod *Japetella diaphana* Hoyle, 1885 were examined using the MBARI Video Annotation and Reference System (VARS)^28^. These species were chosen based on their transparent mantle tissue that allowed for direct observation of gill parasites. *Gonatus* spp. and *V. infernalis* were excluded from the VARS-derived prevalence analysis due to their opaque mantle tissue. Only video segments providing a clear image of the gills were used to quantify parasite prevalence. Prevalence is therefore defined as the percentage of gills infected with parasites of total close-up observations. Suitable video images were selected in reverse chronological order, starting with most recent observations from 2019, to include all prior annotation records until one hundred records for each genus were reached. Despite differences in video resolution over the years, gill parasites could be easily seen on both high (1080 × 1020) and standard definition (640 × 480) video. To estimate prevalence per life stage, we categorized observations as either adult, juvenile or paralarva. In *C. calyx*, life stages can be readily identified by the length the neck and tail, while relative size and body proportions were used to categorize the other host genera^16^. The category ‘adults’ includes both sexually mature individuals and subadults without gonads. The number of parasites per gill was counted to calculate the average infection intensity per specimen, including zeros if one of the gills was without parasites. When only one gill was visible (i.e. in 30 out of 355 observations), average infection intensity per specimen was based on the single gill seen. Finally, using R 3.5.2, prevalence was plotted against depth together with the monthly standardized prevalence (i.e., corrected for the number of ROV dives per month) to investigate spatial and temporal variability.

### DNA extraction

Four different primer pairs suited for protozoan parasites were tested during DNA extractions. These included general eukaryotic 18S rRNA and CO1 primers with two dinoflagellate-specific primers targeting the 18S rRNA region (Supplementary Table S1). The latter primers were included since one of the parasite types matched the description of *Hochbergia moroteuthensis* Shinn & McLean, 1989, a protozoan parasite of unknown taxonomic ranking showing dinoflagellate-like characteristics^29,30^. Out of all four primer pairs, only the primers specifically designed for dinoflagellate cysts^31^ were successful in amplifying *H.* cf. *moroteuthensis’* DNA. DNA was extracted from the parasites with help of the Qiagen DNeasy Blood and Tissue kit (Qiagen Inc., Valencia, CA, USA) according to manufacturer’s instructions. The PCR was then performed using 1 µl of DNA template and 24 µl of master mix, containing 12.5 µl Amplitaq Gold Fast Taq (Applied Biosystems Inc., Foster City, CA, USA), 1 µl forward and reverse primer and 9.5 µl of Milli-Q. For all primers targeting the 18S rRNA region, the cycling program was as follows: 95 °C for 10 min; 35 cycles of 96 °C for 45 s, 53–57 °C for 60 s depending on the annealing temperature of the primers (Supplementary Table S1), 72 °C 90 s; and a final cycle of 72 °C for 10 min. The cycling conditions for the CO1 primers were 95 °C for 10 min; 16 cycles of 96 °C for 10 s, 62 °C for 30 s, 68 °C for 60 s; then 25 cycles of 96 °C for 10 s, 48 °C for 30 s, 68 °C for 60 s; and a final cycle of 72 °C for 10 min. Both the PCR and sequencing amplifications were performed on a Veriti 96 Well Thermal Cycler (Applied Biosystems Inc., Carlsbad, CA, USA).

Amplification was tested using 1.5% agarose/TAE gels, after which the PCR product was diluted in 40 µl of Milli-Q and cleaned with Multiscreen HTS PCR 96 filter plates (Millipore Corp., Billerica, MA, USA). Next, the products were bidirectionally sequenced using the BigDye Terminator v3.1 Cycle Sequencing Kit according to manufacturer’s instructions on an ABI 3500xl Genetic Analyzer (Applied Biosystems, Foster City, CA, USA). The obtained sequences were aligned with dinoflagellate sequences from the curated 18S rRNA database DINOREF^32^ and to the top 100 Blastn matches from the GenBank database, using Geneious 6.0.5 (Biomatters, Auckland, New Zealand) with the MAFFT plugin^33^. To make this comparison more manageable, the DINOREF database was trimmed from 1.671 sequences to 765 sequences by including a maximum of two sequences per species (i.e. 422 dinoflagellate species in total). During the alignment, all database sequences were trimmed to 660 bp to match the length of the obtained parasite sequences. The generated sequences were deposited in GenBank under the accession numbers OL771698-OL771701.

#### Phylogenetic analyses

Phylogenetic trees were constructed based on Maximum Likelihood (ML) and Bayesian Inference (BI) methods. These analyses were done separately for the alignments to the DINOREF and GenBank sequences to avoid cluttering caused by the unidentified eukaryote sequences from the Blastn search. This resulted in four phylogenetic trees total. The ML tree was generated with RaxML v8.2.12^34^, which only implements the General Time Reversible (GTR) model of nucleotide substitution and was therefore used together with a Gamma (Γ) model of rate heterogeneity. Non-parametric bootstrapping with 1000 replicates was used to compute nodal support estimations. The best evolutionary model in the BI analyses was estimated using jModelTest2^35^ based on the corrected Akaike Information Criterion (AICc). This resulted in a GTR model with gamma distribution (GTR + Γ) for both the DINOREF tree and the Blastn tree. The BI analyses included two runs of 30.000.000 generations with 4 chains (three heated, one cold), which were run in MrBayes 3.2.7a^36^. Trees were sampled every 1,000 generations with a burn-in length of 10.000.000 generations to calculate posterior probabilities with the remaining trees. Tracer v1.7.1^37^ was used to assess convergence of model parameters. All trees were constructed with help of the CIPRES Science Gateway^38^.

### Ethics declaration

All specimens in this study were collected under valid US State and Federal Scientific Collecting Permits.

## Results

### Microscopy and molecular identification

**Figure 1:**
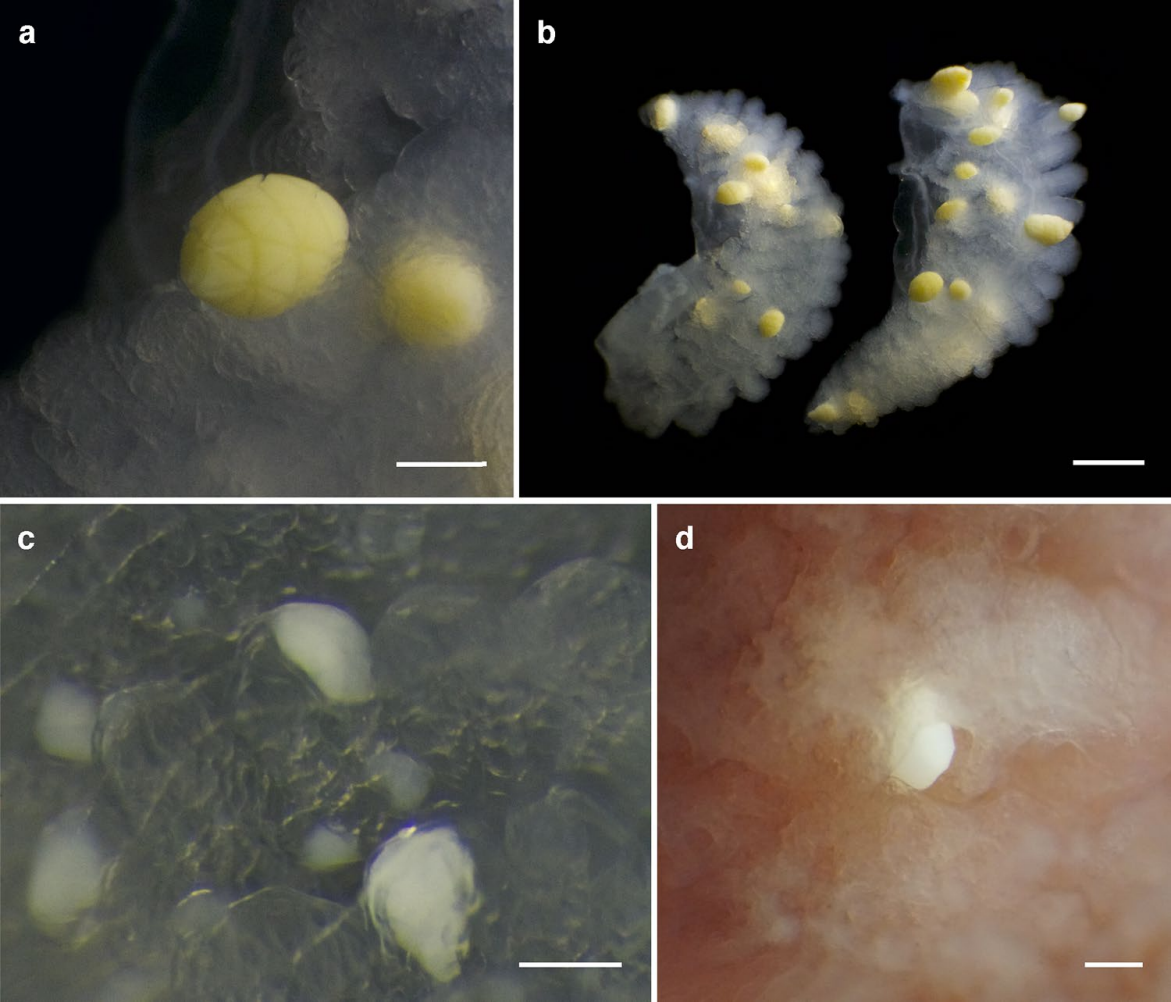
Gill parasites found in three genera of deep pelagic cephalopods. (**a**) *Hochbergia* cf. *moroteuthensis* cyst on tertiary gill lamellae of *Chiroteuthis calyx,* with characteristic triangular thecal plate pattern*.* (**b**) *Hochbergia* cf. *moroteuthensis* covering gill pair of *Chiroteuthis calyx*. (**c**) Parasites from *Gonatus berryi*, embedded within tertiary gill lamellae. (**d**) Parasite from *Vampyroteuthis infernalis*, partially embedded within secondary and tertiary gill lamellae. Scale bars are 0.2 mm in **a**, **c** and **d** and 2.0 mm in **b**.

Based on microscope observations, two distinct parasite types could be distinguished. Two adult specimens of *Chiroteuthis calyx* and two adult *Gonatus berryi* possessed large, ovoid (0.5–1.4 mm long), yellow parasites, covered by an intricate pattern of triangular plates, surrounded by deep grooves (Fig. 1a). These parasites were distributed throughout the gills, attached externally to the primary, secondary and tertiary lamellae (Fig. 1b), and could easily be disassociated with a needle. Their unusual morphology matched the previously described, cyst-like, protistan parasite, *Hochbergia moroteuthensis* of unresolved taxonomic affinity and described off the Oegopsid squid *Onykia robusta* (Verrill, 1876)^29,30^. Since the parasites observed here were found on different hosts and were slightly smaller than the *H. moroteuthensis* type specimens (1.19–1.99 mm)^30^, they will be referred to as *H.* cf. *moroteuthensis*. It was suggested that *H. moroteuthensis* could be of dinoflagellate origin due to the presence of protrusible rod-shaped structures beneath the cell membrane, also known as trichocysts, and an apical pore similar to those found in dinoflagellate cysts^29^.

**Figure 2:**
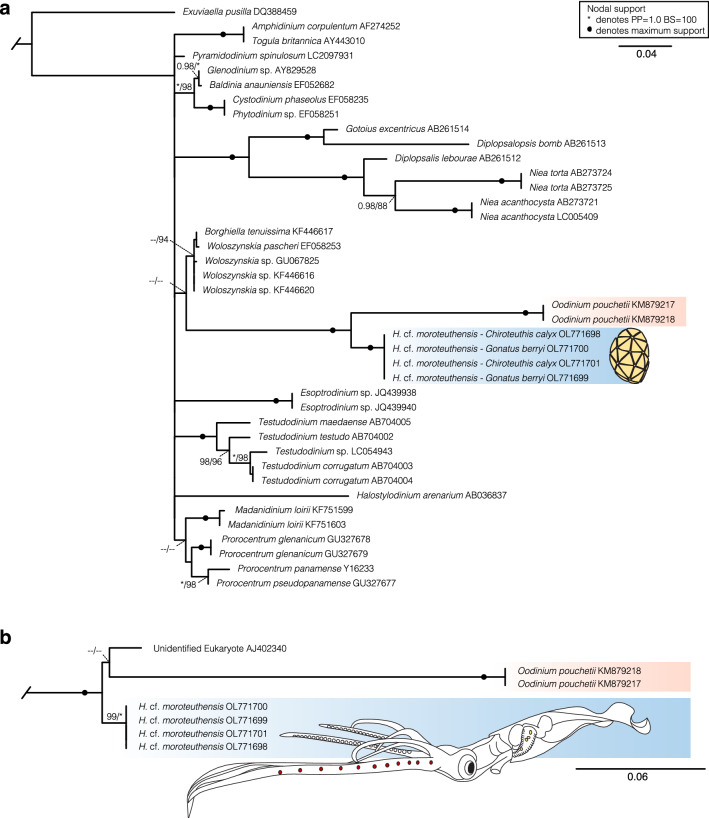
Maximum Likelihood (ML) phylogeny of *H.* cf. *moroteuthensis* 18S rRNA (blue), showing taxonomic affinities to select sequences from the (**a**) DINOREF database^32^ and (**b**) GenBank Blastn matches. Parasitic taxa are highlighted with color blocks, including *Oodinium pouchetii* from the larvacean *Oikopleura* sp. (orange)^41^. The DINOREF tree was trimmed from 794 to 40 sequences to better show details of the larger monophyletic clade comprising *H.* cf. *moroteuthensis* in addition to the outgroup. The Blastn tree was trimmed to show only the smallest monophyletic clade with 90–98% genetic similarity between sequences. Nodes show posterior probabilities (PP)/ bootstrap (BS) values from the Bayesian Inference and ML analyses, respectively. Low support or no values (> 0.95 pp or > 70% bs) are indicated with two dashes (–).

**Figure 3:**
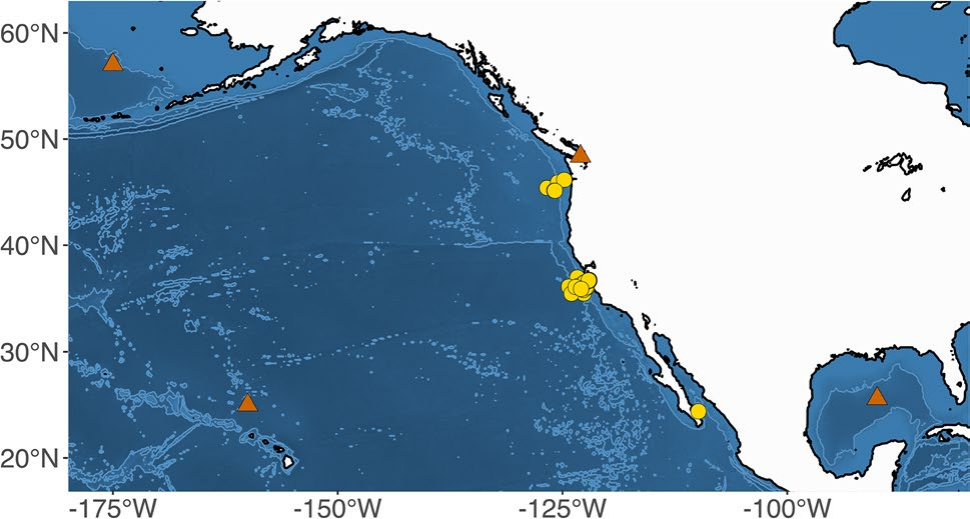
Geographic distribution *H. moroteuthensis*. Orange triangles indicate previously reported distribution in the Bering Sea, Gulf of Mexico and North Pacific Ocean near Washington and Hawai’i^29,30,40^. NB The latter locations are only indicators of reported observations and do not represent actual coordinates as these were not provided in the referenced literature. Yellow circles triangles represent in situ observations of *H.* cf. *moroteuthensis* during MBARI ROV dives on gills of *Chiroteuthis*, *Galiteuthis*, *Taonius* and *Japetella.* Map was generated in R 3.5.2 (https://www.r-project.org).

The partial 18S rRNA sequences obtained for *H.* cf. *moroteuthensis* on *Gonatus berryi* and *Chiroteuthis calyx* were identical. Phylogenetic comparison to the DINOREF dinoflagellate database^32^ confirmed *H.* cf. *moroteuthensis’* placement within the dinoflagellate class Dinophyceae (Fig. 2a). The Bayesian Inference (BI) and Maximum Likelihood (ML) analyses produced congruent results, and recovered *H.* cf. *moroteuthensis* as a monophyletic clade (i.e. 1.0 posterior probability/100% bootstrap support), while *Oodinium pouchetii* (Lemmermann) Chatton, 1912 was recovered as a sister clade with maximum node support. Sequences between these two reciprocally monophyletic clades showed 88% genetic similarity. It should be noted that not all nodes could be resolved using the BI and ML methods, with a large number of clades collapsing onto a polytomy (Fig. 2, Supplementary Figure S1). When comparing *H.* cf. *moroteuthensis* to the top GenBank Blastn matches, the parasites formed a monophyletic clade with *O. pouchetii* and an unidentified eukaryote, collected from the water column at 75 m in the equatorial Pacific Ocean (i.e. environmental sample AJ402340; Fig. 2b)^39^. This environmental sequence showed 97.74% genetic similarity to *H.* cf. *moroteuthensis*. Our finding of *H.* cf. *moroteuthensis* is consistent with the species’ reported distribution: off the coast of Hawaii, the eastern North Pacific Ocean, Gulf of Mexico and Bering Sea (Fig. 3)^29,30,40^.

The second parasite type found in *Gonatus* spp*.* and *V. infernalis* could not be matched to records in the literature. Although parasites from both genera were similar in size (0.2–0.6 mm long), shape and color, their location in the gill suggests they might not be of the same species. In *Gonatus* spp., the parasites appeared to reside within the tertiary lamellae (Fig. 1c), while the parasites in *V. infernalis* were only partly embedded between either the secondary or tertiary lamellae (Fig. 1d). Moreover, the live parasites from *Gonatus* spp. were always white in color, whereas those found in *V. infernalis* occasionally had a light yellow hue. Nevertheless, neither parasite possessed marked structural characteristics that allowed for a definite distinction between the two or narrowed down their taxonomic classification. Both parasites from *Gonatus* spp*.* and *V. infernalis* were relatively easy to remove from the gill tissue with help of a needle. This second parasite type co-occurred with *H.* cf. *moroteuthensis* in two specimens of *Gonatus berryi*.

Molecular analysis failed to provide an identification of the latter parasites. The general eukaryotic primers only amplified host DNA and the dinoflagellate primers produced low quality sequences. In spite of this, we obtained a short 79 bp sequence (i.e. out of the 634 bp total) for the parasites in *V. infernalis* by using the dinoflagellate cyst-specific primer^31^*.* Based on comparison with the GenBank database, this short sequence appears to be from an apicomplexan protist, showing 100.0 percent similarity to select sequences from the genera *Cryptosporidium* Tyzzer, 1907*, Babesia* Starcovici, 1893 and *Theileria* (Bettencourt et al*.,* 1907).

### Prevalence and infection intensity

**Figure 4:**
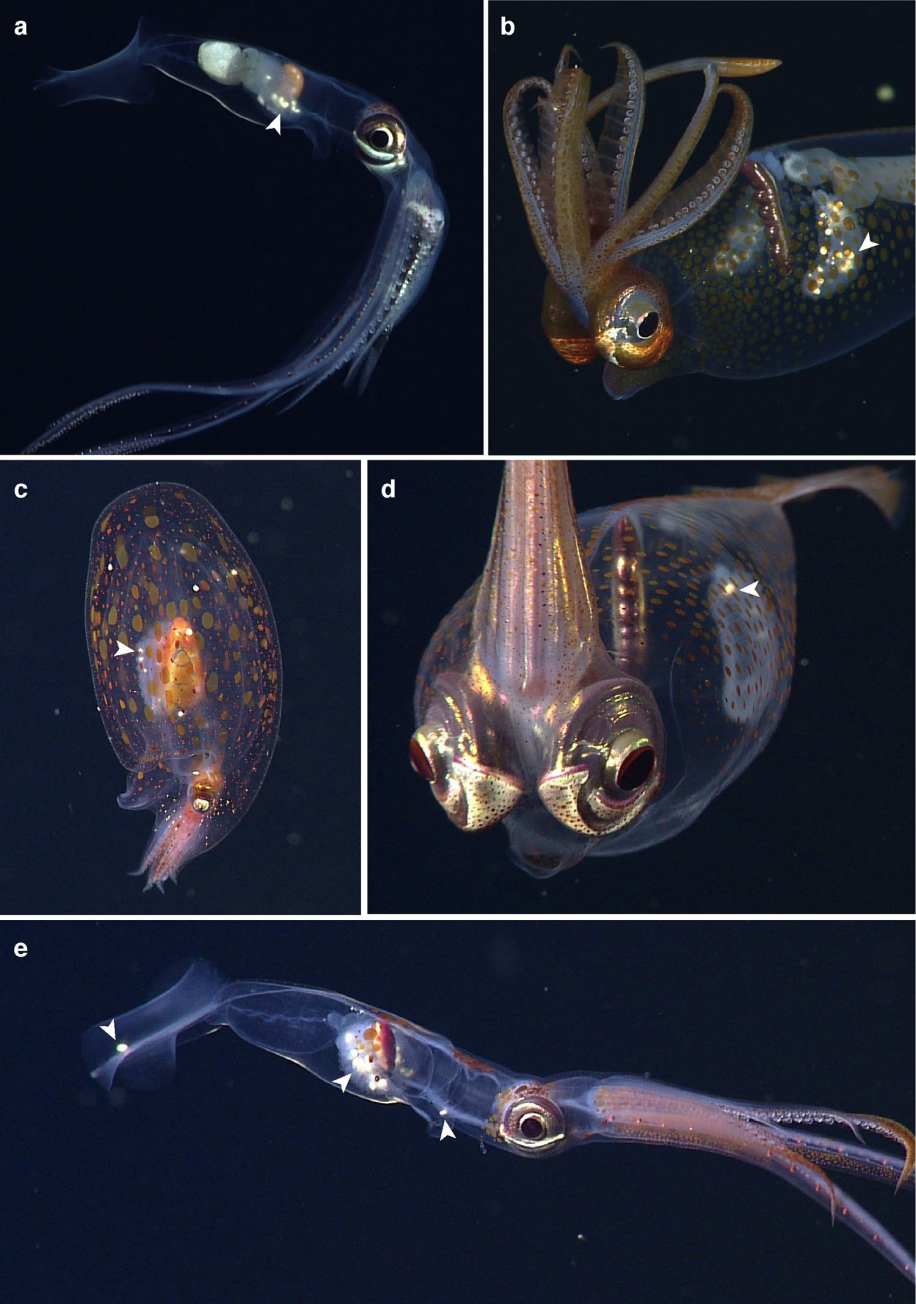
In situ observations of *Hochbergia* cf. *moroteuthensis* on gills of midwater cephalopods (white arrows). (**a**) *Chiroteuthis calyx* Young, 1972, here seen feeding on a myctophid fish. (**b**) *Galiteuthis phyllura* Berry, 1911. (**c**) *Japetella diaphana* Hoyle, 1885. (**d**) *Taonius boreali*s (Nesis, 1972). (**e**) *Chiroteuthis calyx* with unusual infections on the inside of its funnel (right) and upper midline of the fins (left).

Of all the parasite types found in the ROV-collected specimens, a VARS-derived prevalence and infection intensity is given only for *Hochbergia* cf. *moroteuthensis*. Reasons for this were the relatively large size and color of *H.* cf. *moroteuthensis*, making them contrastingly different from other particles on the gills such as marine snow. Nevertheless, since we did not investigate their morphology in close detail, we want to emphasize that gill parasites in this section refer to *H. moroteuthensis*-like parasites. Of the four cephalopod species investigated, *Chiroteuthis calyx* was most commonly infected with *H.* cf. *moroteuthensis* (Fig. 4a, Table 1). All 55 adults of this squid species were infected with parasites, while 86% of the juveniles and 37% of the paralarvae were infected. *Galiteuthis* and *Taonius* had intermediate prevalence of gill parasite (29 and 27%, respectively; Fig. 4b, d), while *Japetella* was only rarely infected (7%, Fig. 4c; Table 1). For the latter genera, adults were the only life stages harboring *H.* cf. *moroteuthensis*, with juveniles devoid of any visible gill parasites. It should, however, be noted that juveniles were only rarely observed and no sightings were available for paralarvae. Observations of *Taonius* and *Japetella* were relatively rare, resulting in fewer video recordings available than initially planned for the prevalence estimate (i.e. 94 and 61, respectively; Table 1). As a consequence, we had to look farther back in the video data base than was necessary for *Chiroteuthis* and *Galiteuthis* (i.e. back to 2001 for *Taonius* and to 1994 for *Japetella*; Table 2)*.*

**Table 1:** Prevalence estimate of *H.* cf. *moroteuthensis* in midwater cephalopods, as the percentage of video observations where gills were clearly visible. n_total_, number of video observations for each taxon; n_sub_, number of video observations per life stage.

Genera	Total %		Adult %		Juveniles %		Paralarvae %	
*Chiroteuthis*	75.0	n_total_ = 100	100	n_sub_ = 55	85.7	n_sub_ = 7	36.8	n_sub_ = 38
*Galiteuthis*	29.0	n_total_ = 100	33.3	n_sub_ = 87	0	n_sub_ = 13		
*Taonius*	26.6	n_total_ = 94	27.2	n_sub_ = 92	0	n_sub_ = 2		
*Japetella*	6.6	n_total_ = 61	6.8	n_sub_ = 59	0	n_sub_ = 2

**Table 2:** Average infection intensity of *H.* cf. *moroteuthensis* per gill of ROV-observed specimens. n, number of gills with parasites; SD, standard deviation; 95% CI, confidence interval.

Genera	Years	Average no. of parasites per gill		SD	95% CI
*Chiroteuthis*	2013–2019	3.26	n = 117	1.90	2.92–3.61
*Galiteuthis*	2013–2019	4.00	n = 51	2.64	3.28–4.72
*Taonius*	2001–2019	1.86	n = 29	1.87	1.18–2.54
*Japetella*	1994–2019	4.00	n = 4	4.08	0.00–8.00

**Figure 5:**
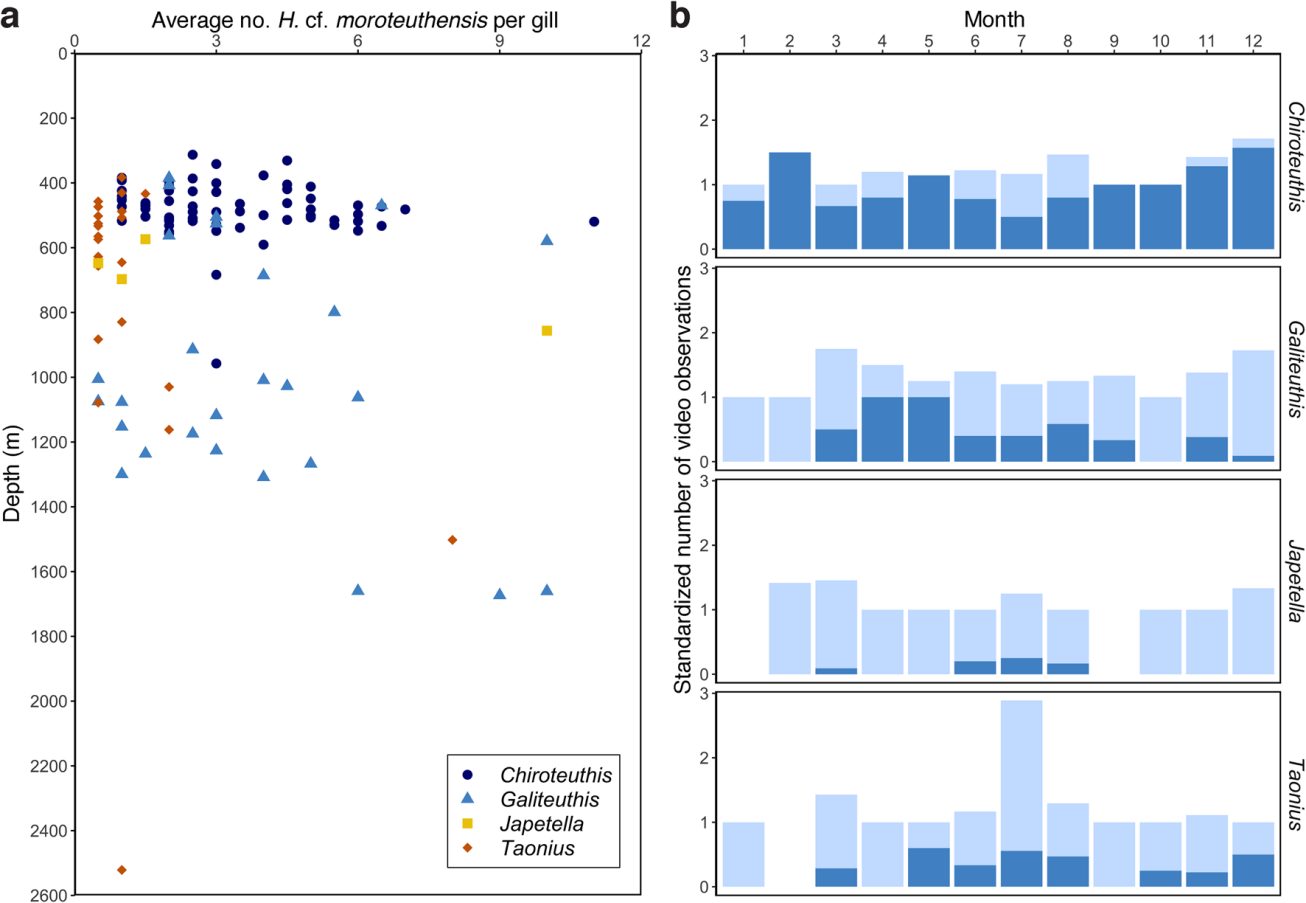
Infection intensity of *Hochbergia* cf. *moroteuthensis* by depth and month. (**a**) Average number of *Hochbergia* per gill per depth for *Chiroteuthis* (blue circle), *Galiteuthis.* (light blue triangle), *Taonius* (orange diamond) and *Japetella* (yellow square). (**b**) Number of cephalopod sightings per month (light blue) parasitized by *Hochbergia* (dark blue). Monthly values are corrected for the total number of ROV dives each month, pooling all recorded years. The numbers 1 to 12 represent the months January to December. Blank entries indicate no host sightings in these months despite dive efforts.

Infection intensity, measured as the average number of parasites per gill, if present, was similar for *Chiroteuthis*, *Galiteuthis* and *Japetella*, ranging between 3.26 and 4.00 parasites per gill with overlapping 95% confidence intervals (Table 2). The infection intensity in *Taonius* averaged only 1.86 *H.* cf. *moroteuthensis* per gill, which was considerably lower than in *Chiroteuthis* and *Galiteuthis* (Table 2). *Hochbergia* cf. *moroteuthensis* was most prevalent in cephalopods between 300 and 600 m (Fig. 5a), which includes the principal depth range of our *Chiroteuthis* encounters, while the overall depth distribution of infected cephalopods ranged between 201 and 2521 m. Even though *H.* cf. *moroteuthensis* was always seen to reside on gills, there was one instance (i.e. out of 355) where two cysts were observed on the inside of the funnel and upper middle of the fin on *C. calyx* (Fig. 4e).

When looking at the variation in gill parasite prevalence over time, parasitized individuals of *Chiroteuthis* and *Galiteuthis* were observed each year during the investigated time period (from 2013 to 2019; Supplementary Figure S2). No yearly trends could be distinguished for either host, with *Chiroteuthis* showing consistently high gill parasite prevalence and *Galiteuthis* relatively low infection numbers. In contrast, parasite prevalence in *Japetella* was more sporadic and parasitized individuals were only observed in four years between 1994 and 2019. For *Taonius* there appeared to be an increasing trend across years, where parasitized individuals were more commonly observed between 2011–2017 compared to 2001–2010 (with no data for 2018 and 2019 due to an absence of observations despite dive efforts). When comparing monthly parasite prevalence, *Chiroteuthis* was parasitized throughout the whole calendar year, January to December (Fig. 5b). *Galiteuthis, Taonius* and *Japetella*, on the other hand, seemed to show more seasonal variation, with an increase in prevalence in spring and summer (i.e. March-03 to August-08, Fig. 5b). Additionally, *Galiteuthis* and *Taonius* showed a slight increase in *H.* cf. *moroteuthensis* infections towards the end of the year (i.e. November-11 and December-12).

## Discussion

Parasites have frequently been observed on the gills of coleoid cephalopods during ROV dives in the mesopelagic waters of the Monterey Submarine Canyon. Here, we demonstrate that at least two parasite species can be distinguished from ROV-collected specimens. Based on morphology, the first parasite was identified as the protist *Hochbergia* cf. *moroteuthensis*. Although the original description of *H. moroteuthensis* struggled to assign a taxonomic rank, the authors noted that the presence of trichocysts and an apical pore bear similarities to those of dinoflagellates in an encysted life stage^29,30^. Using Sanger sequencing and dinoflagellate cyst-specific primers, we confirm this parasite to be a dinoflagellate that forms a sister group to members of the *Oodinium* genus. The second parasite could not be matched to any documented morphological descriptions, and DNA barcoding was only able to resolve a short sequence that does not provide for a reliable identification.

*Hochbergia moroteuthensis* appears to be a common parasite of midwater cephalopods and has previously been collected off the gills of twenty cephalopod species^29,30^. These include five taxa investigated here (*C. calyx*, *V. infernalis*, *Galiteuthis* spp., *Gonatus* spp. and *Japetella diaphana*), with *Taonius* sp. new to the list. While *H.* cf. *moroteuthensis* found in this study was somewhat smaller than the type series (0.5–1.4 mm versus 1.19–1.99)^30^, it was within the range of those reported by McLean et al.^29^ on the squids *Stigmatoteuthis dofleini* Pfeffer, 1912 and *Abralia trigonura* Berry, 1913 (i.e. 0.56 to 1.10 mm on average in length)^29^. The latter authors noticed that parasite size, color (i.e. white to yellow) and thecal plate morphology may differ between host species, which could indicate multiple *Hochbergia* species. It should, however, be noted that it is unknown whether *H. moroteuthensis* maximum growth is dependent on host size or whether the investigated parasites were simply in different growth stages given the study’s relatively small samples sizes. Although we did not compare *H.* cf. *moroteuthensis* morphology across hosts in great detail, the partial 18S rRNA sequences obtained for parasites on *Gonatus berryi* and *Chiroteuthis calyx* were identical. Further research is therefore warranted to investigate species-specific parasite differences and speciation among hosts.

The genetic relatedness between *H.* cf. *moroteuthensis* and its *Oodinium* sister group is further supported by several morphological features. First, the lack of distinct dinoflagellate characters, ovoid shape and the presence of trichocysts, have also been noted for *Oodinium* cysts^41–43^. McLean et al.^29^ further reported that the nucleus of the single-celled *H. moroteuthensis* cyst contains diffuse chromatin, a feature unlike most dinoflagellates that possess well-defined rod-like chromosomes^42^. Remarkably, dinoflagellates within *Oodinium* are known to alternate between both non-dinokaryotic and dinokaryotic nuclei within their life cycles, which could explain *H. moroteuthensis*’ diffuse chromatin^42,43^. Similarities between *H. moroteuthensis* and *Oodinium* further extend to the parasitic life style with primarily pelagic hosts. Dinoflagellates in the *Oodinium* genus are all known to be ectoparasitic, infecting ctenophores, chaetognaths, annelids, larvaceans and a hydromedusa^41,43–46^.

In spite of these similarities, there are also several noteworthy morphological differences between *H. moroteuthensis* and members of the *Oodinium* genus. Young *Oodinium* cysts generally have a white to yellow coloring, with older cysts taking a yellow–brown or dark brown tint^41,43,44^. *Oodinium* cysts also possess relatively simple thecal plates and above all, have a distinct peduncle, or stalk, with which they attach to the host and which is thought to serve as feeding apparatus^41,43,47^. Maximum lengths for *Oodinium* cysts have been reported up to 0.39 mm^43,46^. In contrast, cysts in *H. moroteuthensis* possess a white to yellow coloring, an intricate pattern of triangular plates, reach sizes up to 1.99 mm long, and have a simple holdfast area with an oval aperture that likely anchors them to the host^30^. Currently, both *Oodinium* and *Hochbergia* form a genetically distinct clade within the Dinophyceae and analysis of further specimens and genetic markers might provide more insight into their relatedness and specialization on primarily pelagic hosts. Additionally, analysis of fast- and slow-evolving genetic markers might resolve the polytomy observed in the phylogenetic trees, which were also present in the phylogenetic reconstruction of the DINOREF reference database by Mordret et al.^32^.

**Figure 6:**
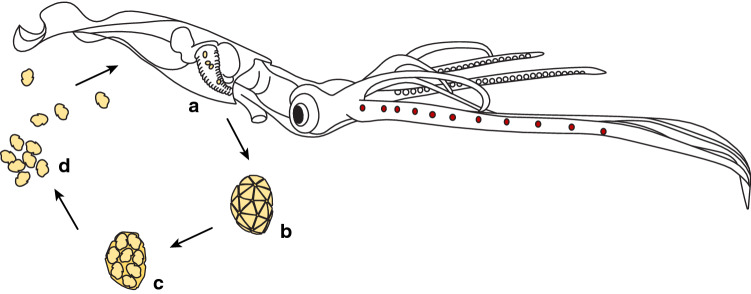
Theorized life cycle of *Hochbergia moroteuthensis*. (**a**) The vegetative trophont (feeding life stage) grows without cellular division on the cephalopod’s gills. (**b**) The mature trophont detaches and (**c**) divides into motile dinospores, (**d**) free to infect new hosts in the water column. Illustration (**b**) trophont adapted from Shinn & McLean^30^.

The genetic similarity of *H.* cf. *moroteuthensis* to an unidentified eukaryote from the water column and the fact that we encountered the protozoans in an encysted stage, strongly suggests that these dinoflagellates infect their cephalopod hosts through a free-living life stage. Many parasitic dinoflagellates, including *Oodinium,* alternate between a motile free-living stage—the dinospore—that forms a vegetative feeding stage—the trophont—upon attachment to the host^41,47,48^. During this vegetative stage, the trophont grows greatly in size but without cellular division. Once mature, the trophont detaches from the host to divide into multiple flagellated dinospores. The dinospores disperse into the water column, free to infect new hosts (Fig. 6)^41,47,48^.

Such a free-living life stage is consistent with *H. moroteuthensis*’ wide geographic distribution. Free-living dinospores are easily dispersed by ocean currents, and observations in both the North Pacific Ocean and the Gulf of Mexico could indicate large-scale ocean connectivity, potentially beyond the distribution reported here^29^. This dispersal may also offer *H. moroteuthensis* a wide range of infection possibilities and explain why trophonts are found in twenty-one different cephalopod taxa. Nevertheless, population genetic structure needs to be investigated, as it is currently unknown if the parasites represent multiple species.

Free-living dinospores might also explain *H. moroteuthensis*’ location on the exterior gill tissue. With dinospores free in the water column, the fastest pathway to a cephalopod’s interior is through ‘inhalation’. In this process, cephalopods actively force water through their gills, making these the first organs *Hochbergia* would encounter. Respiratory organs give direct access to the cephalopod’s blood stream, and therefore offer a suitable environment (i.e. nutrient and oxygen rich) for development into a trophont. Gills also provide interstices that could simply trap dinospores. Either way, there was only one occasion (i.e. out of 355) where trophonts were seen on other body parts besides the gills (Fig. 4e). In comparison, several *Oodinium* parasites are also known to attach to specific host-body parts, apparently preferring sites involved in locomotor movement. For instance, *Oodinium jordani* McLean & Nielsen, 1989 is known to attach to the fin of the chaetognath *Sagitta elegans* Verrill, 1873^46^, while *O. pouchetti* is mostly found on the tail of appendicularians^41^, and *Oodinium* sp. collected off various ctenophores appears to prefer attachment close to or within the beating comb rows^44^. Whether these surface areas offer highest encounter rates or provide a physical benefit such as enhanced oxygenation remains unknown.

The increased prevalence of *H.* cf. *moroteuthensis* observed in the most abundant cephalopod, *Chiroteuthis,* and in the other adult cephalopods is in line with infection dynamics known from other wildlife parasites, where the probability of a parasitic infection increases with host density and age^49–51^. Following this, dinospores in the Monterey Submarine Canyon have more opportunities to encounter common squids like *Chiroteuthis*^52^ and longer-lived cephalopods. Alternatively, it is possible that the increased parasite load in adults is simply the result of larger gill surface areas when compared to juveniles. However, when comparing prevalence between host species, it should be noted that the maximum adult sizes for *C. calyx* (up to 100 mm in mantle length, ML) are smaller than those of *Galiteuthis* (500 mm ML)*, Taonius* (660 mm ML) and *Japetella* (144 mm ML) among specimens found in the Monterey Submarine Canyon^53,54^*.*Other factors that might explain the observed prevalence include parasite preferences for host physiology (e.g. respiration rates) or confinement to a certain depth range^18^. Although *Chiroteuthis*, *Galiteuthis, Taonius* and *Japetella* partially overlap in their depth distributions, *Chiroteuthis* generally remains above the core of the oxygen minimum zone, located around 700 m in Monterey Bay^52,55^. *Galiteuthis*, on the other hand, has a bimodal distribution, with older individuals known to migrate below the oxygen minimum core^52,55,56^. If dinospore viability is restricted to more shallow depths, the probability of infection for *Galiteuthis* could decrease when living at deeper depths. This is further supported by *Taonius*, which showed a comparable bimodal distribution to *Galiteuthis*^52^ and shared a similar parasite prevalence. Furthermore, *Japetella* is the deepest living cephalopod investigated and harbored relatively few *Hochbergia* trophonts. In spite of this, it is unknown how long it takes for *H. moroteuthensis* dinospores to develop into mature trophonts and over what time frames they may accumulate on their hosts. Lab-based experiments with *Oodinium* sp. on the ctenophore *Beroe abyssicola* Mortensen, 1927 showed that trophonts needed approximately 20 days to grow from 35 µm in length to their mature size of 350 µm at 10 °C^44^. Given that *H. moroteuthensis* can grow over five times larger and lives at colder temperatures depending on its host distribution, growth periods may be substantially longer.

When looking at the prevalence of *H.* cf. *moroteuthensis* over time, only *Taonius* appeared to be showing an increase in infected individuals over the years. Present results, however, are insufficient to determine whether this increase is the result of environmental change or part of natural variability. We therefore recommend continued monitoring to determine long term trends. Based on the monthly prevalence, it is likely that *Chiroteuthis* acts as a reservoir for *Hochbergia* parasites throughout the year. *Galiteuthis, Japetella* and *Taonius* show more seasonal dynamics. It may be that the reported seasonality is related to upwelling events or environmental cues promoting dinospore formation (e.g. increasing temperatures)^50^. Alternatively, cephalopods might be more susceptible to infections in certain months, or have higher resistance in others. *Taonius*, for example, had a markedly lower parasite load on average than *Galiteuthis* despite similar prevalence estimates (Tables 1 and 2), potentially indicating some sort of resistance mechanism. More research is warranted to confirm any host resistance and the influence of depth or seasonal effects.

The other parasite type found in ROV-collected specimens of *Vampyroteuthis infernalis* and *Gonatus* spp. needs further characterization. Although DNA barcoding was able to resolve a short sequence that potentially places it within the phylum Apicomplexa, it appears more likely that this genetic material originated from contamination with a different parasite. Apicomplexa reported in cephalopods generally infect the digestive tract and are morphologically different from the parasites observed here^19^.

In conclusion, our findings highlight the need for further investigation of cephalopods and their gill parasites. Considering that parasites influence biodiversity and that cephalopods form key links in pelagic food webs, future research should be focused at assessing potential effects on cephalopod physiology. For example, if *H. moroteuthensis* limits longevity or reproduction in common squids like *C. calyx*, then changes in parasite abundance might result in cascading effects on abundance of *Chiroteuthis*’ prey, predators and competitors. Additionally, baseline estimates of parasite prevalence are crucial to fully understand whether midwater host-parasite systems are at risk from increasing anthropogenic stressors and how they will change over time. While ROV observations have proven key to estimate prevalence and infection intensity here, trawled specimens continue to be valuable for verification of parasite species and obtaining material for genetic analyses, even if slightly damaged. We therefore recommend combining ROV observations with periodic trawling in future studies, since ROVs may not reveal smaller parasites, early infections or parasites in animals with tissue that is not transparent.

## Supplementary Information

Supplementary Information.

## Data Availability

Generated sequences for *H. moroteuthensis* were deposited in GenBank under the accession numbers OL771698-OL771701.
