# Supplementary material for: ROV observations reveal infection dynamics of gill parasites in midwater cephalopods
Source: Sci Rep. 2022 May 18;12:8282. doi: 10.1038/s41598-022-11844-y (PMC9117243; doi:10.1038/s41598-022-11844-y)
Supplement: Supplementary file 1 — Supplementary Information. [file 41598_2022_11844_MOESM1_ESM.pdf]

ROV observations reveal infection dynamics of gill parasites in midwater cephalopods

Vanessa I. Stenvers[1,2*], Rob E. Sherlock[3], Kim R. Reisenbichler[3], Bruce H. Robison[3]

[1] GEOMAR, Helmholtz Centre for Ocean Research Kiel, Düsternbrooker Weg 20, 24105 Kiel, Germany

[2] Department of Invertebrate Zoology, National Museum of Natural History, Smithsonian Institution, Washington, DC 20013, U.S.A.

[3] Monterey Bay Aquarium Research Institute, 7700 Sandholdt Road, Moss Landing, CA 95039-9644, U.S.A.

*Corresponding author: Vanessa I. Stenvers (vstenvers@geomar.de)

Keywords: ectoparasitism, Cephalopoda, *Hochbergia,* deep sea

SUPPLEMENTARY FIGURE

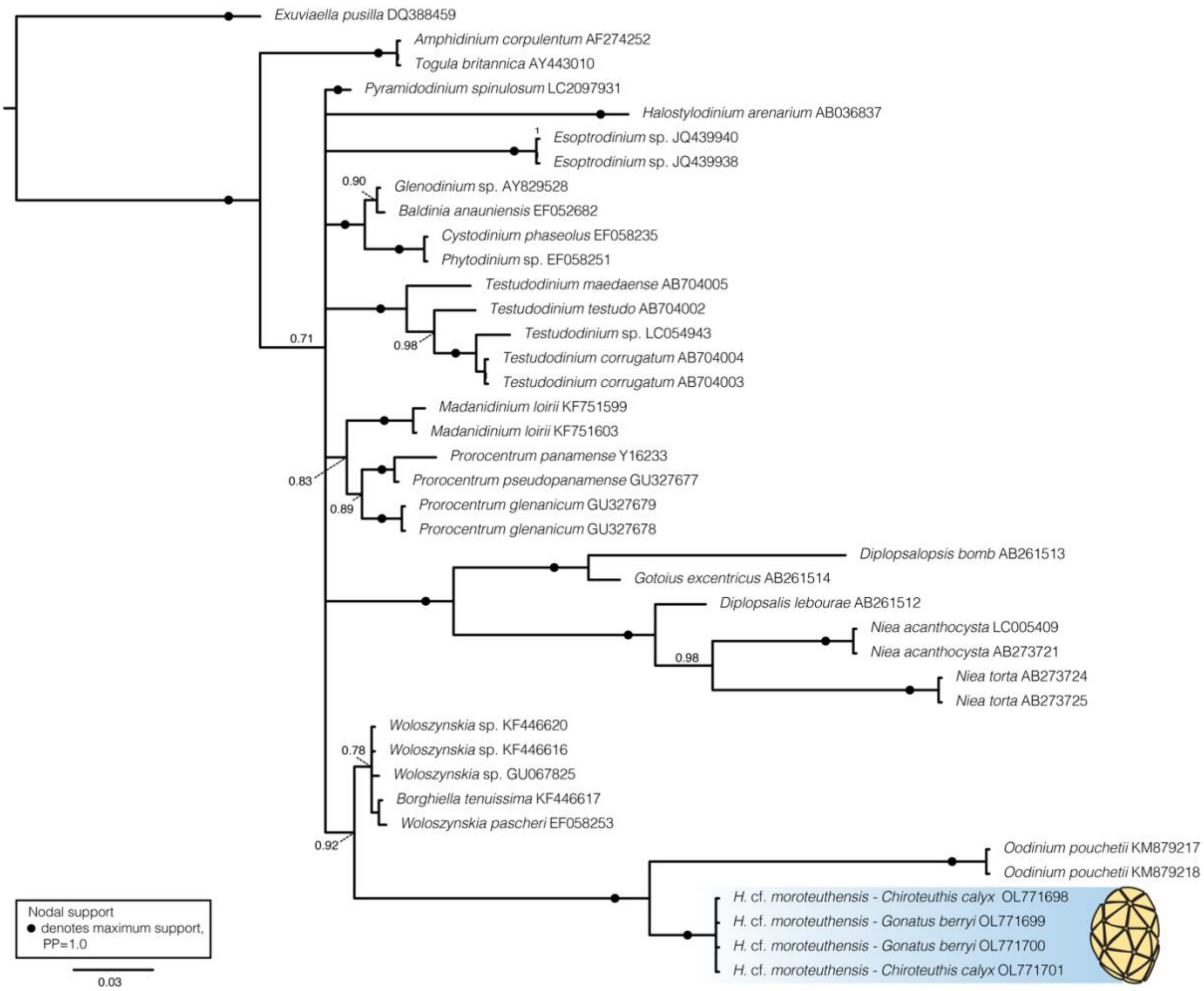

Supplementary Figure S1. Bayesian Inference (BI) phylogeny of *H.* cf. *moroteuthensis* 18S rDNA (highlighted in blue) and select sequences from the DinoREF database[1]. The tree was trimmed from 794 to 40 sequences. Nodes show posterior probabilities (PP).

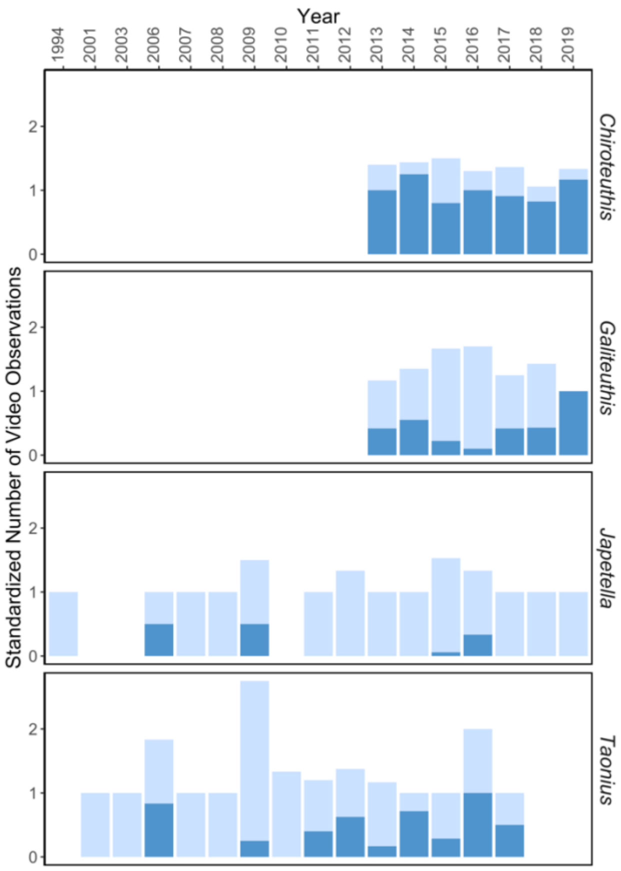

Supplementary Figure S2. Number of cephalopod sightings per year (light blue) parasitized by *Hochbergia* cf. *moroteuthensis* (dark blue) as seen during ROV dives. Yearly values are corrected for the total number of ROV dives each year. Prevalence for *Chiroteuthis* and *Galiteuthis* was investigated between 2013–2019, for *Taoinius* between 2001–2019, and for *Japetella* between 1994–2019. Any blank entries within the investigated time ranges indicate no host sightings in those years, despite dive efforts.

SUPPLEMENTARY TABLE

Supplementary Table S1. Primers used for molecular identification of the cephalopod gill parasites. Each primer pair is listed in the order of forward and reverse with T_a indicating the annealing temperature. ('Euk' indicates eukaryotic primers and 'Dino' dinoflagellate-specific ones).

**Target region**	**Primer**	**Sequence (5' – 3')**	**T_a (°C)**	**Reference**
Euk 18s rRNA	1391F	GTACACACCGCCCGTC	57	[2]
	EukBr	TGATCCTTCTGCAGGTTCACC		
Euk CO1	LCO1490	GGTCAACAAATCATAAAGATATTGG	48	[3]
	HC02198	TAAACTTCAGGGTGACCAAAAAATCA		
Dino 18s rRNA	18ScomF1	GCTTGTCTCAAAGATTAAGCCATGC	53	[4]
	Dino18SR1	GAGCCAGATRCDCACCCA		
Dino cyst 18s rRNA	18S634F	GGGTAACGGAGAATTAGGGTTT	53	[5]
	18S634R	TCCCCTAACTTTCGTTCTTGATC		

REFERENCES

1 Mordret, S. *et al.* dinoref: A curated dinoflagellate (Dinophyceae) reference database for the 18S rRNA gene. *Molecular Ecology Resources* **18**, 974-987, doi:10.1111/1755-0998.12781 (2018).

2 Maritz, J. M. *et al.* An 18S rRNA workflow for characterizing protists in sewage, with a focus on zoonotic trichomonads. *Microb Ecol* **74**, 923-936, doi:10.1007/s00248-017-0996-9 (2017).

3 Folmer, O., Black, M., Hoeh, W., Lutz, R. & Vrijenhoek, R. DNA primers for amplification of mitochondrial Cytochrome C oxidase subunit I from diverse metazoan invertebrates. *Molecular marine biology and biotechnology* **3**, 294-299 (1994).

4 Lin, S., Zhang, H., Hou, Y., Miranda, L. & Bhattacharya, D. Development of a dinoflagellate-oriented PCR primer set leads to detection of picoplanktonic dinoflagellates from Long Island Sound. *Appl Environ Microbiol* **72**, 5626-5630, doi:10.1128/AEM.00586-06 (2006).

5 Gao, Y. *et al.* An improved method for the molecular identification of single dinoflagellate cysts. *PeerJ* **5**, e3224-e3224, doi:10.7717/peerj.3224 (2017).
